# Management of perioperative low cardiac output state without extracorporeal life support: What is feasible?

**DOI:** 10.4103/0974-2069.74045

**Published:** 2010

**Authors:** Girish Kumar, Parvathi U Iyer

**Affiliations:** Department of Pediatric and Congenital Heart Surgery, Fortis Escorts Heart Institute, New Delhi, India

**Keywords:** After load reduction, cardiopulmonary interactions, extracorporeal life support, low cardiac output state, low cost strategy, lusitropy, rescue therapy, restrictive physiology

## Abstract

A transient and reversible reduction in cardiac output–low cardiac output state (LCOS) often occurs following surgery for congenital heart disease. Inappropriately managed LCOS is a risk factor for increased morbidity and death. LCOS may occasionally be progressive and refractory needing a period of “myocardial rest” with extracorporeal life support (ECLS). ECLS is currently considered a routine tool available for rapid deployment in most industrialized countries. Accumulated experience and refinements in technology have led to improving survivals – discharge survivals of 35%–50%, with almost 100% survival in select groups on elective left ventricular assist device. Thus, there is an increasing trend to initiate ECLS “early or electively in the operating room” in high-risk patients. India has a huge potential need for ECLS given the large number of infants presenting late with preexisting ventricular dysfunction or in circulatory collapse. ECLS is an expensive and resource consuming treatment modality and is not a viable therapeutic option in our country. The purpose of this paper is to reiterate an anticipatory, proactive approach to LCOS: (1) methods for early detection of evolving LCOS and (2) timely initiation of individualized therapy. This paper also explores what is feasible with the refinement of “simple, conventional, inexpensive strategies” for the management of LCOS. Therapy for LCOS should be multimodal based on the type of circulation and physiology. Our approach to LCOS includes: (1) intraoperative strategies, (2) aggressive afterload reduction, (3) lusitropy, (4) exclusion of structural defects, (5) harnessing cardiopulmonary interactions, and (6) addressing metabolic and endocrine abnormalities. We have achieved a discharge survival rate of greater than 97% with these simple methods.

## INTRODUCTION

### Perioperative low cardiac output state

A transient and often reversible reduction in cardiac output–low cardiac output state (LCOS) with an associated decrease in systemic oxygen delivery often occurs following surgery for congenital heart disease.[1,2] The LCOS if not recognized in time and managed appropriately may be “progressive” leading to multi - organ dysfunction, increased morbidity, prolonged ICU and hospital stay, and even mortality.

### Why does LCOS occur?

The reduction in cardiac output is due to a transient “myocardial dysfunction” following cardiopulmonary bypass (CPB).[1] Factors implicated in the development of myocardial dysfunction include: (1) the intense inflammatory response associated with CPB, (2) myocardial ischemia from prolonged aortic cross-clamping, (3) inadequate myocardial protection, (4) reperfusion injury, (5) hypothermia and (6) large ventriculotomies when performed. Further reductions in cardiac output occur due to residual or undiagnosed structural lesions or in instances of late presentation with preexisting right ventricular, left ventricular or biventricular dysfunction.[3]

### Risk factors for perioperative LCOS

The risk is greatest for neonates and young infants undergoing complex surgical repairs, those needing prolonged aortic cross clamp times, those presenting in circulatory collapse and those infants and children with preexisting right ventricular, left ventricular or biventricular dysfunction.[3]

### Extracorporeal life support in the industrialized world: A routine and useful tool

Extracorporeal life support (ECLS) is currently considered a routine and useful tool in the pediatric cardiac intensive care unit and is available for rapid deployment whenever needed in most industrialized countries. Current indications for ECLS include: (1) failure of separation from cardiopulmonary bypass, (2) postoperative severe low cardiac output state or failed hemodynamics, (3) postoperative cardiac arrest, (4) severe pulmonary vascular hypertension and (5) acute respiratory distress syndrome.[4] ECLS has been used postoperatively in both bi-ventricular repairs (commonest being–arterial switch, anomalous origin of the coronary artery (ALCAPA), tetralogy of Fallot) as well as in single ventricular situations.[5]

Refinements in ECLS have led to steadily improving outcomes with discharge survivals of 38% in neonates and 43% in older children.[6,7] Factors improving odds of survival were: (1) early initiation of ECLS in the operation theatre than in the cardiac intensive care unit (64% survival vs 29%) and (2) use of ECLS for severe reactive pulmonary hypertension.[8] Alsoufi *et al*. reported an impressive overall hospital survival of 67% for ECLS after surgery for congenital heart disease and a 100% survival in select subgroups with elective or early use of ventricular assist device (VAD) for single ventricle and biventricular disease.[5] Further refinements in the ICU with the use of “rapid cardiopulmonary support” as compared to conventional ECMO have improved the 30-day survival to ~65% in children with failed hemodynamics.[9]

Thus, with accumulated experience and refinements in technology the trend in the industrialized world is to initiate ECLS “early or even electively in the operating room” rather than as a “desperate late rescue modality.”[5,8] Currently, many units feel that about 3%–8% of infants undergoing surgery for congenital heart disease may benefit from early institution of perioperative ECLS. Today, some western units also believe that ECLS may actually be cost saving–reducing ventilation and ICU stay.[10]

### ECLS in India: A potential need, high cost, and unavailability

The potential need for ECLS in India is “huge” given the large numbers of infants with transposition of great arteries, obstructed total anomalous pulmonary veins, truncus arteriosus presenting late, i.e., with unstable hemodynamics or with severe reactive pulmonary hypertension. Infants with “preexisting ventricular dysfunction” undergoing definite surgery – (ALCAPA, late d-transposition of great arteries with intact ventricular septum [dTGA. IVS]) are on the increase–constituting a potential and undisputed substrate of infants who are likely to benefit from elective or early institution of ECLS.

In reality, ECLS is not freely available in most parts of the non-industrialized world where according to current western recommendations it is possibly most needed. Why is this so? The reasons are primarily the prohibitive costs, lack of infrastructural resources as well as the highly skilled and well trained manpower that an ECLS program entails. In our country, despite the rapid industrialization over the last decade, pediatric cardiac care is mainly provided by “nongovernmental institutions.” Thus, the costs of ECLS have to be largely borne by the family. Most cases of refractory LCOS, where ECLS may be most useful often occur in infants and children whose families are least able to afford such expenses. Despite favorable cost–utility analysis in the West,[10] many hospitals and other sponsors feel that the costs are too prohibitive and that “that kind of money” can be used for the benefit of many more children.

The second practical consideration is the complex circuitry with a “very narrow margin for error” which has the potential to increase dangerous complications in the hands of staff of widely varying capability.[11] Western literature has also shown that the current favorable results of ECLS have been associated with a “definite learning curve.”

Thus, in our country, alternative, reproducible, less expensive modalities which are “not resource consuming” assume increasing importance and often need to be speedily employed. The various strategies used for “perioperative manipulation of the circulation” in children with congenital heart disease have been elegantly summarized in a recent article by Shekerdemian.[12]

The purpose of this paper is to reiterate some simple, conventional, evidence based, low cost strategies for the management of perioperative LCOS that are practiced in our unit.

### Pediatric cardiac surgery without ECLS back up: What is feasible?

Even though progressive LCOS occurs after cardiac surgery, appropriate anticipation, early identification and aggressive management has been shown to minimize the need for ECLS to <2% of all children who undergo CPB.[1] Thus, our current multipronged approach is proactive and focuses on “simple, evidence based, and affordable measures to diminish requirement of mechanical support” in high risk surgeries.

We have been successful with this approach. Our annual 30 day survival for the last 7 years has been in the range of 98%–98.5% in patients with mean Basic Aristotle risk stratification score ranging from 6.9–7.0. The annual 30 day survival for the last 7 years for neonates ranges from 91% to 94% and annual 31 day to 1-year old survival (the sickest group which includes infants of late presentation, severe pulmonary hypertension and severe right, left or biventricular dysfunction) is 97%–98.5%.

### What did we do to achieve these outcomes without “rescue” ECLS?

**Figure 1:**
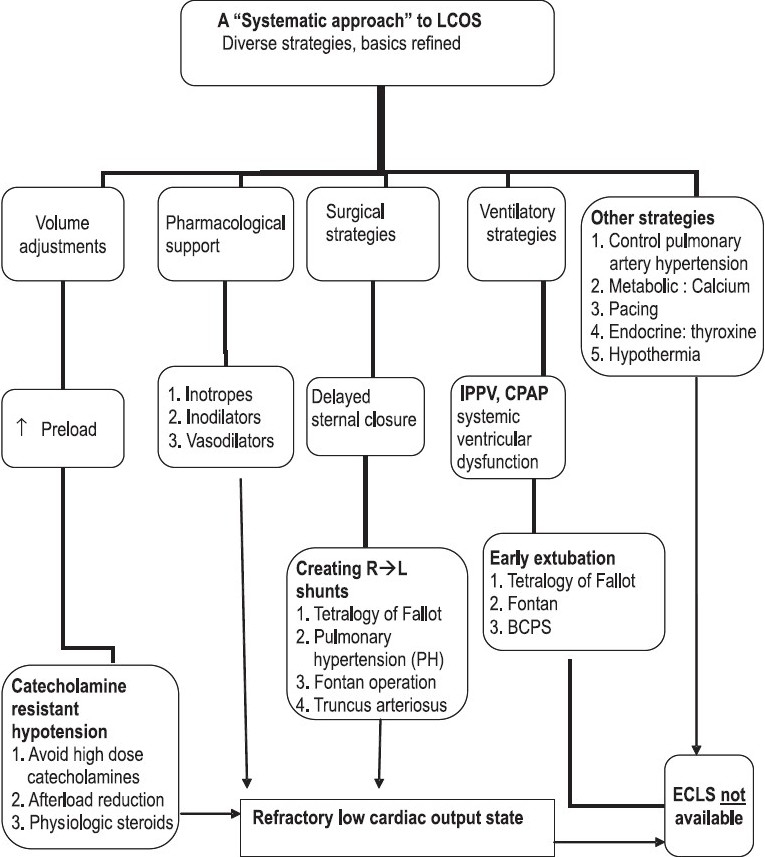
LCOS management without ECLS: A multimodal approach

We follow a “systematic approach” to LCOS based on refinement of a range of diverse conventional strategies illustrated in Figure 1.

#### a) Surgical strategies

Our focus is on efficient surgery with (1) shorter aortic cross clamp times and (2) avoiding long ventriculotomies to minimize postoperative LCOS. We practice well-described preemptive surgical strategies like creation of a “right to left shunt or a decompressive patent foramen ovale” as a “pop off” mechanism for the hypertensive right ventricle (select cases of infant tetralogy of Fallot, severe pulmonary hypertension, e.g., hypertensive VSDs, hypertensive truncus arteriosus). Likewise, there has been a lower threshold for “deferred sternal closure” in the operating room to avoid emergency sternotomies in the ICU. Our indications for deferred sternal closure include: ventricular dysfunction, myocardial edema, high left atrial pressures, and late presenting total anomalous pulmonary venous connections.

An important preemptive surgical strategy in our unit is the use of routine intraoperative echocardiography to refine surgical repairs and to avoid significant residual defects. In a review of children on postoperative ECMO, 60 cardiac catheterizations resulted in a total of 50 transcatheter, surgical or combined interventions.[13] This paper reiterates that residual structural defects constitute an important cause of perioperative LCOS[13]– a cause that can be readily prevented.

We have also evolved a useful preemptive surgical strategy of leaving a “residual dynamic right ventricular outflow tract gradient” after repair of late presenting tetralogy of Fallot to minimize postoperative right ventricular dysfunction and secondary LCOS.[14]

#### b) Other intraoperative measures: (use of corticosteroids, ultrafiltration, phenoxybenzamine)

We use various well-described modalities to minimize CPB mediated injury in an attempt to reduce perioperative LCOS and ensure better hemodynamics.[15–19]

This includes the use of prophylactic intraoperative corticosteroid administration to attenuate the CPB related intense inflammation.[15–18] Shroeder *et al*. showed that combined preoperative and intraoperative corticosteroid administration was more effective in diminishing inflammatory mediator expression and was associated with better systemic oxygen delivery.[15] A “best evidence” article[17] on the subject concluded that intraoperative steroids reduce C reactive protein (CRP), interleukin 6 and troponin I after CPB but evidence for “clinical benefit” is only limited. A recent systematic review concluded that there is “weak favorable evidence” for prophylactic steroid therapy.[18] Our practice is to routinely use intraoperative methyl prednisolone in all infants (under 1 year of age, <10 kg) and whenever possible use “combined preoperative and intraoperative methylprednisolone” in neonates and small infants undergoing complex surgery.

Ultrafiltration (conventional, modified, and combined) has been shown to be effective in the (1) removal of inflammatory mediators – (interleukin 6 and tumor necrosis α) as well as in the (2) reduction of extracellular total body water secondary to CPB with improvement in cardiac and pulmonary function.[19,20] We use conventional ultrafiltration in all infants under 1 year of age, those <10 kg or in sick children with preoperative cardiac failure.

Intraoperative phenoxybenzamine–a potent vasodilator had fallen into disrepute over the last many years due to the prolonged vasodilation, associated hypotension and absence of an effective antidote. There has been a resurgence of interest in phenoxybenzamine in recent times.[21] Apart from the intense α (alfa) blockade, phenoxybenzamine helps in organ protection during deep hypothermic CPB and improves postoperative systemic oxygen delivery.[12,22] Our center favors the use of intraoperative phenoxybenzamine (prior to CPB) in a select group of patients–all neonates, very small infants, all left to right shunts under 1 year of age or less than 10 kg and severely pulmonary hypertensive left to right shunts at any age. Phenoxybenzamine is continued in the ICU in the dose of 1–2 mg/kg/day.

#### c) Failure to wean from cardiopulmonary bypass–our strategy

Cardiac ECMO for biventricular repairs is instituted in about 60% of instances for “failure to wean” satisfactorily from CPB.[8] Our strategy in this instance is to search assiduously for a residual defect by a meticulous intraoperative echo.[12–13] Once residual defects have been satisfactorily excluded, partial CPB is used to offload the heart in cases of ventricular dysfunction (late arterial switch, ALCAPA) or unstable hemodynamics (elevated left atrial pressures [LAp] in late obstructed total anomalous pulmonary venous connection [TAPVC]).

#### d) Early recognition of “evolving low cardiac output state” and impaired systemic oxygen delivery

A typical time course for “CPB related LCOS” has been shown by Wernovsky *et al*. with the nadir in cardiac output occurring 9–12 h afte surgery–the cardiac output approaching “normal” within 24 h.[23] An evolving low cardiac output state should always be suspected if the postoperative course is unexpected.[12] Our focus is to diagnose an “evolving LCOS” rather than a florid established low output state–which may be harder to manage.

A combination of clinical, hemodynamic and biochemical parameters is used to diagnose “evolving LCOS.”[12,24,25] Clinical features include tachycardia, systemic hypertension or hypotension, core hyperpyrexia, cool peripheries, reduced toe temperatures and unexpected agitation despite adequate sedation and analgesia. Persistent elevations in central venous, right atrial or left atrial pressures are important hemodynamic parameters suggestive of inadequate myocardial function. Intermittent or persistent ventricular or supraventricular arrhythmias unrelated to electrolyte imbalance are strongly indicative of ventricular dysfunction and should be urgently investigated, rather than merely treated.

Biochemical markers of impaired systemic oxygen delivery include increased metabolic acidosis, elevated blood lactate levels and reduced mixed venous oxygen saturation. Worsening metabolic acidosis is a relatively late sign of reduced cardiac output, therefore, an attempt has to be made to diagnose LCOS before frank metabolic acidosis develops. Elevated blood lactate level during the first 8 hours of surgery has been shown to be a useful predictor of adverse hemodynamic events in infants and children operated for heart disease and has been used for adjustment of hemodynamic support.[26] Evolving or early LCOS may be associated with normal lactate level and a combination of lactate and mixed venous oxygen saturation (SvO_2_) has been shown to be more useful in predicting evolving LCOS.[27] A fall in SvO_2_ to below 55% has been associated with increased early morbidity and mortality.[28]

Blood lactate level and mixed venous oxygen saturation are performed routinely on arrival in the ICU. It is our practice to upgrade the inotropic support if the SvO _2_ is less than 70% or if the arterio venous oxygen saturation difference (SaO_2_–SvO_2_) is greater than 30% in systemic to pulmonary artery shunts and cavo pulmonary connections (bidirectional Glenn, Fontan operations).[24] More importantly, “trends” in hemodynamic parameters and biochemical markers of adequacy of oxygen delivery are more useful than absolute values. These are carefully monitored following upgrading of inotropes and in the absence of improvement, patients are investigated further.[12,25]

Apart from intraoperative echos, serial echos are also done in the unit to detect subclinical systolic or diastolic ventricular dysfunction, patch dehiscence, residual defects and pericardial collection.

#### e) Principles of postoperative LCOS management

Successful management involves more than “inotropy.”[12,24,25] The management of LCOS is based on a multi pronged strategy addressing the inflammatory, metabolic and hormonal causes of myocardial dysfunction. Systolic and/or diastolic dysfunction needs to be diagnosed and managed appropriately. Therapy also needs to be “tailored” according to which ventricle is dysfunctional–the management of right ventricular and left ventricular dysfunction being quite different. Ventilatory adjustments are increasingly used as a “hemodynamic tool” with better understanding of cardiopulmonary interactions in different circulations.[12,24,25]

#### f) Algorithm based inotrope use

Our practice of inotrope and vasoactive medication use is neither empirical nor arbitrary. It is based on an alogorithm, which is periodically modified and refined with emerging evidence. The following are the broad principles, which will provide a template for our current practices.

1. Preload optimization with fluid augmentation before inotropy is of paramount importance in improving stroke volume.[29] Residents and nurses are taught to recognize and treat both “absolute and occult hypovolemia” to improve cardiac output.
2. Our preferred first line inotrope has been dobutamine for the last many years. We do not use dopamine. Our practice is backed by recent data demonstrating the adverse effect of dopamine on systemic hemodynamic status and oxygen transport in infants following the Norwood operation.[30]
3. If additional inotropy is needed, we upgrade to milrinone after weighing “cost benefit” considerations. The Primacorp study (a well-designed prospective randomized controlled study) demonstrated that the use of high dose milrinone reduced the risk of LCOS in a heterogenous group of infants undergoing biventricular repairs.[31] Milrinone is a potent inodilator and a lusitropic agent, without the adverse effects of increased myocardial oxygen consumption, tachyphylaxis, or myocardial apoptosis.
  However, the use of milrinone is strictly individualized and not entirely based on the recommendations of the Primacorp study due to cost constraints. By and large, the neonate, infant or child has to “earn the need for milrinone use.”
  Separation from CPB is usually achieved with dobutamine in most instances-neonates, small infants, arterial switches, TAPVC re-routing, tetralogy of Fallot, large L-R shunts with borderline operability. Milrinone is commenced electively in the operating room only occasionally. Our indications for institution of Milrinone are shown in Table 1.
4. We continue dobutamine when milrinone is added. This practice is based on the observation that appropriate “lusitropy and inotropy are additive” in improving cardiac output.[25]
5. Milrinone is commenced in a very low dose of 0.2 *μ*g/kg/min and gradually graded up in sick hypotensive, very high-risk neonates and infants.[12,32]
6. Inotropes are upgraded (number of drugs and dosage increase) according to various parameters which are closely titrated.[12,25] These parameters are as follows.
  - Clinical: Significant tachycardia, peripheral vasoconstriction.
  - Biochemical: Deranged SvO_2_, SaO_2_–SvO_2_ difference, lactates, anion gap.
  - Hemodynamic: Persistent LaP surges, increased CVP.
  - Echo: Worsening systemic ventricular function, significant pulmonary hypertension with or without RV systolic dysfunction, significant RV diastolic dysfunction
7. Other inotropes: Epinephrine is used only occasionally in our unit. It is used only for resuscitation and occasionally, in low dose, for severe systolic dysfunction. Doses of epinephrine greater than 0.2 mc/kg/min are associated with significantly increased afterload and are generally avoided.[12]
8. Vasopressors: Norepinephrine is used for right ventricular dysfunction especially when associated with significant peripheral vasodilatation, in vasodilatory shock[12] and in postoperative residual dynamic left ventricular outflow tract obstructions. We have no experience with the use of vasopressin.

**Table 1:** Indications for use of milrinone in our unit

In the OR before/for separation from CPB significant systemic ventricular dysfunction; persistent LA surges, e.g., late TAPVC; select cases of tetralogy of Fallot. In the PICU worsening parameters on dobutamine; clinical: significant tachycardia, peripheral vasoconstriction; biochemical: deranged SvO_2_, increasing SaO_2_–SvO_2_ difference/lactates/anion gap; hemodynamic: persistent La surges, e.g., late TAPVC; Echo: significant or worsening systemic ventricular dysfunction Tetralogy of Fallot: usually in PICU if: worsening features of LCOS (clinical, hemodynamic, biochemical, echo); Echo: significant diastolic dysfunction with borderline parameters and extensive repair Rationale: Usefulness in restrictive physiology, safer than dobutamine in residual dynamic RVOTO TAPVC: usually in PICU if worsening features of LCOS (clinical, hemodynamic, biochemical, echo); increasing LA pressures–double digits! Rationale: Smallish, noncompliant LV, nonroomy LA

#### g) Aggressive afterload reduction

**Figure 2:**
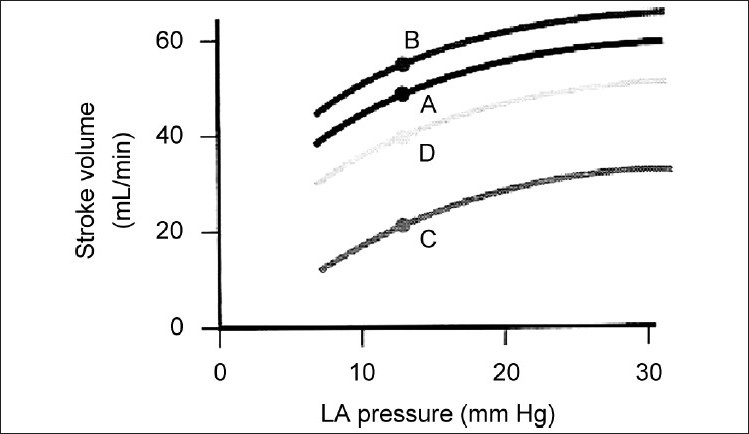
Effect of afterload reduction. Afterload reduction is of greater benefit in severe ventricular dysfunction. Increase in stroke volume with afterload reduction is greater in severe ventricular dysfunction-baseline C moves to D, baseline A to B only

Aggressive afterload reduction is our mainstay of management in severe systolic ventricular dysfunction. This practice is based on the observation that afterload reduction is associated with a proportionally greater increase in cardiac output in severe ventricular dysfunction as compared to mild or moderate ventricular dysfunction.[25] [Figure 2]. Afterload reduction has been shown to be particularly useful to augment stroke volume and overall cardiac output in neonatal hearts as well as in those with poor myocardial contractility.

Afterload reduction in the postoperative period is achieved with:

1. Phenoxybenzamine—commenced intraoperatively and continued postoperatively—the main problem being its long half-life (>24 h) occasionally causing protracted and severe systemic hypotension.
2. Sodium nitroprusside is easier to titrate and is a gentler vasodilator due to its short half-life and rapidity of action.
  Sodium nitroprusside is used for afterload reduction due to its favorable qualities and low cost. It is particularly useful in infants and children with postoperative systemic hypertension associated with normal systemic ventricular function or mild systemic ventricular dysfunction. The dose is titrated to systemic blood pressures and peripheral temperatures.
  Thus, indications in our unit for the use of sodium nitroprusside are: coarctation repair, duct ligation, Fontan operation (it is our drug of choice), valvuloplasty/valve replacements, and occasionally large L-R shunts (isolated VSDs, VSDs + PDA + ASDs, i.e., L-R shunts at multiple levels).
3. Milrinone: In any of the above situations if there is worsening lactic acidosis, increasing SaO_2_–SvO_2_ gradient, increasing peripheral vasoconstriction (exclude occult hypovolemia) or other features of LCOS—(clinical, biochemical, hemodynamic, echo) or significant systemic ventricular dysfunction then the policy is to upgrade to milrinone [Table 1].
  We manage primary left ventricular dysfunction after an arterial switch for late presenting dTGA with aggressive afterload reduction than with increasing inotropy.

#### h) Nitroglycerine

Nitroglycerine is used primarily for its venodilator properties following valvuloplasty or valve replacement and as a pulmonary and coronary vasodilator (coronary spasm). Nitroglycerine is also used for preload optimization whenever the central venous pressures are elevated.[33]

#### i) Arrhythmias-prompt recognition and aggressive management

A “nonsinus rhythm” is associated with a lower cardiac output than a sinus rhythm. Similarly very slow or very rapid heart rates are associated with suboptimal cardiac output. Arrhythmias occur frequently in the postoperative cardiac surgical pediatric patient-the most common being nonsustained ventricular arrhythmias with an incidence of 22%.[34] Every attempt is made to restore sinus rhythm or obtain hemodynamic advantage by appropriate pacing techniques, cardioversion, or pharmacological intervention.[1,24,25,34]

Junctional ectopic tachycardia (JET) is a common and potentially dangerous tachyarrhythmia that tends to occur in the first 48 hours especially after surgeries involving closure of a large ventricular septal defect including tetralogy of Fallot.[35] Younger infants and neonates have a greater predilection for JET. It is very poorly tolerated leading rapidly to very severe LCOS especially if the infant is already hemodynamically unstable. Hypomagnesemia is frequent after open heart surgery and is known to contribute to JET. We routinely check magnesium levels and correct hypomagnesemia in an attempt to reduce the incidence of JET based on published recommendations.[36] Once JET occurs, the goal is to re-establish atrioventricular synchrony in an attempt to rapidly improve cardiac output. Measures used to treat established JET are: (1) discontinue adrenergic agents, e.g., dobutamine if possible,[35] (2) pacing either atrial or atrioventricular sequential, (3) if the rate is very fast pharmacologic therapy with amiodarone,[37] (4) induction of core hypothermia.[25]

Nonsustained ventricular arrhythmias and JET are both worrying arrhythmias and need further evaluation i.e. to look for occult ventricular dysfunction and residual structural defects.

#### j) Severe LCOS: Metabolic and hormonal control–Use of calcium, thyroxine and insulin

Myocardial contraction and relaxation are mediated by cyclic fluctuations in cytoplasmic calcium concentration. The neonatal and infant myocardium depends much more on “extracellular calcium” for its systolic and diastolic function, since the sarcoplasmic reticulum is sparse and immature. Thus, it is important to maintain adequate extracellular calcium levels in neonates and small infants.[38] Hypocalcemia is common in the postoperative neonates,[25] those infants on long term frusemide and in malnourished infants. Our practice is to measure serial ionic calcium levels and maintain them using intravenous infusion.[25,38]

Thyroid hormone levels (triiodothyronine and thyroxine) have been shown to be reduced for prolonged periods after cardiopulmonary bypass contributing to postoperative myocardial dysfunction.[39] Two studies–one small study and one randomized control study showed that triiodothyronine treatment in infants with postoperative LCOS showed improvement in cardiac output.[40,41] Our practice is to measure thyroid function in all postoperative cardiac infants and administer thyroxine to all infants with abnormal thyroid function.

Hyperglycemia in the postoperative cardiac infant has been shown to adversely impact outcomes.[42,43] Our preliminary experience in 302 infants and children showed that hyperglycemia greater than 200 mg/dl increased LCOS and inotrope requirement. However, the concerns about insulin induced severe hypoglycemia in neonates and small infants are real. Our current practice is to treat hyperglycemia greater than 200 mg/dl with a continuous insulin infusion with careful monitoring of blood glucose and potassium levels.

#### k) Postoperative “persistent LCOS”: An assiduous search for mechanical issues is warranted

Residual structural defects should be diligently looked for if the post operative recovery is not “as expected.” Our approach to persistent postoperative LCOS is aggressive – meticulous echo cardiographic evaluation followed by cardiac catheterization if needed.[13,24,25]

#### l) Catecholamine resistant hypotension: Adjuvant management strategies

**Figure 3:**
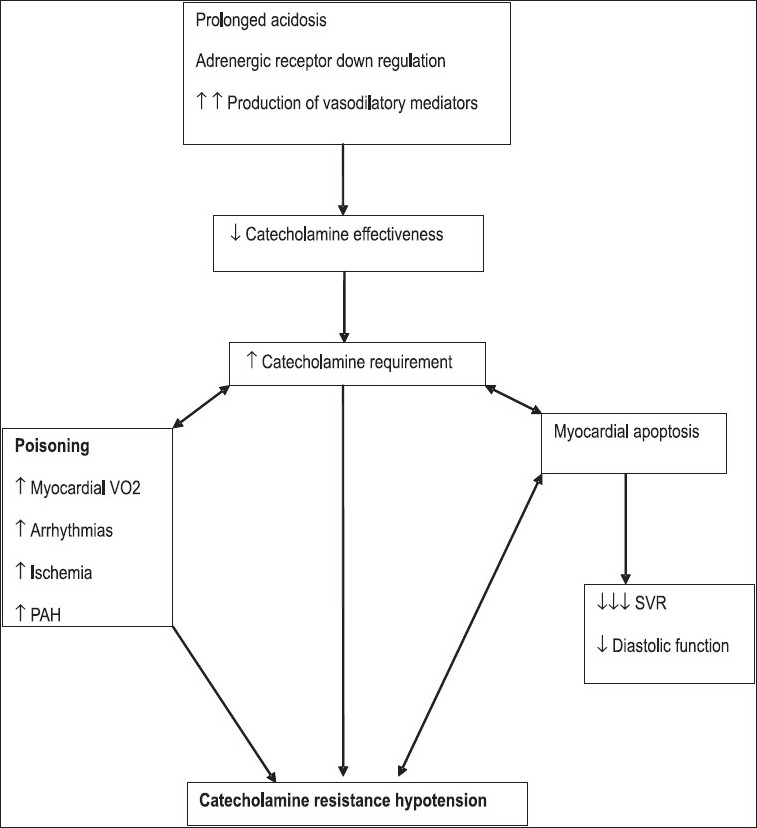
Pathophysiology of catecholamine resistant hypotension: Need for alternative therapies

Prolonged acidosis and high dose catecholamines are known to induce adrenergic receptor down regulation reducing catecholamine effectiveness leading to a vicious cycle of increased catecholamine requirement, increased myocardial oxygen consumption, tachycardia, tachyarrhythmias, increased afterload, myocardial ischemia, apoptosis and aggravation of systolic and diastolic dysfunction giving rise to a scenario of catecholamine resistant hypotension.[44] [Figure 3]

Thus, despite issues of late presentation and pre-existing ventricular dysfunction the policy in our unit is to avoid high dose catecholamines. Published data suggest that seriously ill patients have relative adrenal insufficiency.[45] In a retrospective study of critically ill postoperative cardiac neonates, low dose corticosteroids reduced the epinephrine requirement as well as the inotrope score within 24 hours.[46] We administer hydrocortisone 50 mg/m^2^/day to sick neonates and infants with LCOS. We do not wait for refractory LCOS to develop and have a low threshold to use low dose corticosteroid in high risk scenarios.

#### m) Harnessing cardiopulmonary interactions: Use of judicious positive end expiratory pressure (PEEP) and noninvasive ventilation

Positive pressure ventilation is an important hemodynamic tool in the management of postoperative systolic ventricular dysfunction.[12,25,47]

In a landmark study, Bradley *et al*. demonstrated that the institution of nasal continuous positive airway pressure (CPAP) in adults with cardiogenic shock and increased left ventricular end diastolic pressures significantly improved cardiac output.[48] Positive pressure ventilation and PEEP have several beneficial effects in acute systolic heart failure improving overall cardiac output. Positive pressure ventilation improves the work of breathing, offloads the right ventricle by decreasing right ventricular preload, reduces left ventricular afterload, diminishes endogenous catecholamines and myocardial oxygen consumption-thereby improving “efficient cardiac output”.[47] Positive pressure ventilation and PEEP also help in optimizing alveolar recruitment thereby improving systemic oxygen delivery. Thus, positive pressure ventilation constitutes an important hemodynamic support in postoperative systolic ventricular dysfunction.[12,25,47]

The group of patients who specifically benefit from the hemodynamic effect of positive pressure ventilation include those with post operative systolic systemic ventricular dysfunction: (1) small infants and older children following repair of large left to right shunts (VSDs, VSDs + large PDAs, etc.), (2) primary left ventricular dysfunction following arterial switch for transposition of great arteries, (3) repair of total anomalous pulmonary veins, 4) repair of anomalous origin of the coronary artery, (5) primary left ventricular dysfunction following aortic or mitral valvuloplasty or replacement. Our policy is to ventilate these patients till hemodynamically stable and routinely support them with “elective continuous positive airway pressure (CPAP-nasal or mask)” for a period of time after tracheal extubation.[12,47] We have successfully used “prolonged nasal or mask CPAP” as hemodynamic support even in severe left ventricular systolic dysfunction (LVEF 10%-15%) following surgery for ALCAPA and late presenting large VSDs with aortic valvuloplasty.

#### n) Specific clinical scenarios

##### 1. Tetralogy of Fallot

**Figure 4:**
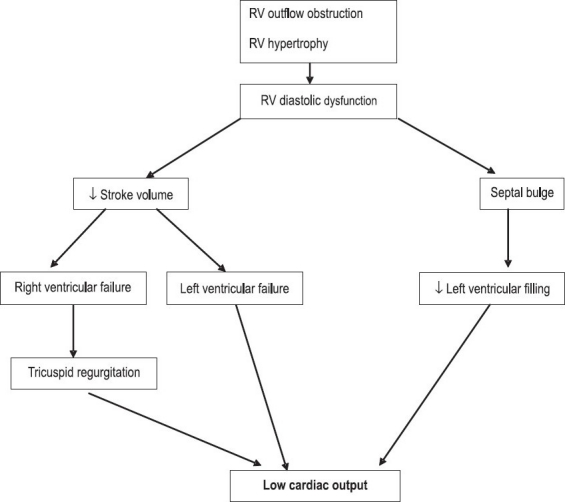
Restrictive physiology in postoperative tetralogy of Fallot

Surgery for tetralogy of Fallot comprises 20%–25% of all surgeries performed for congenital heart disease in India. In the current era, surgery for tetralogy of Fallot is associated with excellent discharge survivals of greater than 98%–99%.[49] Early post operative mortality in tetralogy of Fallot is due to a rapidly worsening early onset LCOS which may very quickly become refractory to conventional therapy. Low cardiac output is due to the underlying restrictive physiology which may be compounded by associated JET.[12,25] In an elegant study Redington *et al*. characterized the right ventricular diastolic performance after complete repair of tetralogy of Fallot.[50] This study showed that these infants have varying degrees of postoperative right ventricular diastolic dysfunction and highlighted the importance of diastolic antegrade pulmonary blood flow in sustaining cardiac output [Figure 4].

Thus, measures to optimize cardiac out in post operative tetralogy of Fallot include those which improve diastolic pulmonary flow. These are a combination of ventilatory and hemodynamic strategies.[50,51] Ventilatory strategies include early extubation and if ventilation is required for an edematous infant then ventilation with “short inspiratory times” is recommended.[50,51] Shekerdemian *et al*. showed the benefits of negative pressure ventilation in improving cardiac output in postoperative tetralogy of Fallot.[52] Negative pressure ventilation is technically cumbersome and may not be feasible in routine clinical practice. However, her work clearly underscores the deleterious effects of prolonged positive pressure ventilation in post operative tetralogy of Fallot and reiterates the importance of establishing spontaneous respiration as soon as possible to optimize cardiac ouput.[51,52] Hemodynamic measures in the management of postoperative tetralogy include: (1) gentle volume augmentation to ensure optimal filling of a “stiff right ventricle, (2) avoidance of excessive catecholamines which may “worsen a residual dynamic right ventricular outflow obstruction” with acceptance of lower blood pressures, (3) prevention and control of tachycardia.[50] Measures to prevent and treat JET have already been discussed earlier.

In summary, our management of tetralogy of Fallot is based on a combination of well described modalities. They are surgical strategies (dynamic residual RVOT gradients, decompressive patent foramen ovale in infants), ventilatory and hemodynamic measures to augment pulmonary diastolic flow and optimize cardiac output. An intraoperative peritoneal dialysis catheter is sited routinely in infants to decompress the abdomen and prevent ascites secondary to right heart failure to facilitate early extubation. Our outcomes with these measures have been gratifying—discharge mortality of 0.8% in a consecutive series of 900 surgeries which include complex tetralogy of Fallot.

##### 2. Fontan operation:

The Fontan operation is characterized by a passive forward pulmonary blood flow which depends on an adequate preload and a low resistance unobstructed pulmonary circulation. The pulmonary blood flow in Fontan patients depends on a low intrathoracic pressure. Thus, forward pulmonary blood flow and cardiac output is optimal during spontaneous respiration and significantly compromised by positive pressure ventilation.[12,47,53–55] Thus, our practice is to avoid sedation in post operative Fontan patients and extubate them as soon as possible on arrival in the ICU.[55]

##### 3. Bidirectional cavo–pulmonary shunt

The circulation in the bidirectional cavo-pulmonary shunt (BCPS) is similar to the Fontan in that the pulmonary blood flow is preload dependent and depends on a low resistance pulmonary circuit. Since the pulmonary blood flow is entirely derived from the upper body venous return any elevation in superior vena caval pressures, increase in pulmonary artery pressures or increase in airway resistance adversely affect pulmonary flow, cardiac output and cause systemic desaturation.

Our practice is to nurse infants coming to the ICU after a BCPS in the sitting position to assist systemic venous drainage and augment forward pulmonary blood flow.[12,25] We ventilate without heavy sedation and accept modest hypercarbia (pCO_2_ in ~high 40s) to enhance cerebral vasodilatation and reduce superior vena caval pressures. Any reactive airways is aggressively managed and the infant is extubated as soon as possible.[12] These proactive measures have been shown to optimize systemic oxygen delivery and improve hemodynamics after BCPS in recent studies.[56–58]

#### (o) Clinical bottomline: A multimodal “individualized” approach recommended

In summary, a multimodal “highly individualized” approach based on simple, inexpensive evidence-based methods is recommended for management of perioperative LCOS. We have had satisfying outcomes with this approach without rescue ECLS in two very difficult situations-primary arterial switch for the late presenting dTGA with IVS and ALCAPA with left ventricular dysfunction.

Primary outcomes (in hospital mortality 4.5%) and secondary outcomes (ventilatory duration 72 hours, hospital stay 11.5 days, organ dysfunction) were comparable in a group of 22 consecutive infants who underwent late primary arterial switch (median age 55 days) to the early switch group.

Similarly, excellent results were obtained in 16 consecutive infants who underwent repair for ALCAPA with left ventricular dysfunction. In-hospital mortality was 0, with a median ventilation of 5 days and hospital stay of 15.5 days using conventional methods without rescue ECLS.

#### (p) Where do we go from here? Is our system perfect ?

Over the years, we have learnt to refine standard therapies and individualize LCOS treatment to specific circulations without resorting to rescue ECLS. Deaths do occur due to refractory LCOS in our unit, but they are occasional and usually related to lack of recognition of (1) evolving LCOS, (2) acute pulmonary hypertension, (3) dangerous arrhythmias, and (4) failure to detect a residual defect.

Thus, our current focus is more on “fast track training” of new recruits and “better error control” than the pursuit of ECLS.

## CONCLUSION

Many western units have moved to a “routine ECLS policy” in select sub groups.[59] We need to have our own “low cost practical approach to LCOS”. A highly pre-emptive approach to perioperative LCOS is recommended to minimize post operative morbidity, mortality, and need for ECLS. Early identification and aggressive treatment of LCOS along with an assiduous search for residual structural defects is central to the successful management of perioperative LCOS. Additional lessons we learnt over the years are to avoid “flogging a sick myocardium” with excessive catecholamines but to focus on (1) preload optimization, (2) appropriate afterload reduction, (3) lusitropy, (4) harnessing cardiopulmonary interactions (use of various ventilatory strategies as a powerful hemodynamic tool), and address (5) metabolic and endocrine abnormalities contributing to LCOS.
